# Gestational tumors as a model to probe reticulate evolution in human neoplasia

**DOI:** 10.18632/oncotarget.26510

**Published:** 2019-01-08

**Authors:** Yuri Lazebnik

**Affiliations:** ^1^ Lerna Consulting, New Haven, CT, USA

**Keywords:** cancer, reticulate evolution, gestational tumors, cell fusion, horizontal gene transfer

## Abstract

Reticulate evolution, which involves the transfer of genes and other inheritable information between organisms, is of interest to a cancer researcher if only because “pirating” a trait can help a cell and its progeny adapt, survive, or take over much faster than by accumulating random mutations. However, despite being observed repeatedly in experimental models of neoplasia, reticulate evolution is assumed to be negligible in human cancer primarily because detecting gene transfer between the cells of the same genetic background can be difficult or impossible. This commentary suggests that gestational tumors, which are genetically distinct from the women who carry them, provide an opportunity to test whether reticulate evolution affects the development of human neoplasia.

The evolutionary model of cancer views neoplastic cells as unicellular asexual organisms that evolve by accumulating genetic and epigenetic alterations [1-5]. Accordingly, cancer development is represented as a phylogenetic tree, akin to the tree of life that depicts the evolution of species [2, 6, 7].

The tree of life, however, has been increasingly resembling a net, as genome sequencing has revealed that organisms of all kingdoms, and unicellular organisms in particular, can transmit their genes not only vertically, from parents to offspring, but also horizontally, from one organism to another [8-12]. Such speciation, which involves not only branching, as depicted by the tree of life, but also gene transfer, hybridization, symbiosis, and other mechanisms of information exchange, is fittingly called reticulate evolution, from a Latin word for “having a net-like pattern” [13].

Reticulate evolution is of interest to a cancer researcher because “pirating” a trait by gene transfer or having a trait emerge as a result of hybridization can help an organism and its progeny adapt, survive, or take over much faster than by accumulating random mutations. For example, a gene transfer that happened between two fungi eight decades ago - an instance on the evolutionary timescale - resulted in a virulent wheat pathogen which has since spread worldwide [14]. Even complex traits can be acquired within a generation, which for unicellular organisms and cells means days, if not hours.

The possibility of reticulate evolution in neoplasia is especially intriguing because some of its mechanisms, including horizontal gene transfer and hybridization between neoplastic or normal cells, have been documented to affect or even define carcinogenesis and tumor progression in experimental models and proposed to do so in human disease [15-21]. This proposal, however, is rarely considered in designing cancer treatment or prevention because the studies of reticulate evolution in human neoplasia are scant and their results inconclusive.

The primary reason for this fact is that documenting DNA transfer or hybridization is difficult or impossible if the genomes of the donor and recipient cells cannot be distinguished by sequencing, genotyping, or karyotyping. A chromosome acquired from a neighboring cell becomes indistinguishable from a chromosome inherited through an error in mitosis, and a cell hybrid from a cell that duplicated its genome by skipping cytokinesis.

In the laboratory, this problem is routinely and unambiguously solved by using chimeric animals, engineering reporter genes, or by grafting tumors derived from one species into another.

In humans, reticulate evolution has been probed by analyzing tumors arising in organ transplant recipients, who have cells of two genomically distinct individuals in their bodies, their own and those of the donor [22-24]. However, these patients and their samples have proven to be too rare to provide a critical mass of evidence required to make definitive conclusions or encourage a deeper inquiry.

A more accessible yet overlooked alternative, which I would like to suggest, is to analyze gestational neoplasia, which are tumors resulting from pregnancy. The remarkable feature of these tumors is that they are genomically foreign to the patient because they arise from her fertilized eggs [25-27].

Normally, a fertilized egg develops into a fetus, which has a copy of both the paternal and maternal genomes. However, at the incidence that varies worldwide between 20 and 1300 per 100,000 pregnancies [26], a fertilized egg develops into a lesion, the hydatidiform mole. Some of the moles, called partial, have both the maternal and paternal genomes, while others, known as complete, have only the paternal. About 15% of moles, mostly complete, become invasive and about 5% metastasize [28].

The most aggressive gestational tumor, however, is choriocarcinoma, whose incidence varies between 2 and 200 cases per 100,000 pregnancies [26]. Half of these cancers result from a normal pregnancy and have both maternal and paternal genomes, while the other half stem mostly from complete moles and thus carries only the paternal genome [25], an ideal condition for studying gene exchange or hybridization between the tumor and host cells.

Gestational tumors have several advantages over tumors arising in transplant patients.

First, gestational tumors are more accessible. While finding cancer patients who had a prior organ transplant requires some serious investigative work and results only in one to a few cases reported in each of the few published studies [24], a recent study reported a set of tissue samples and circulating tumor cells obtained from 115 gestational carcinoma patients [29].

Second, because gestational tumors are genomically foreign to the patient, they can be used to detect gene exchange with any cell type of the body. In transplant patients, such exchange can be detected only with the cells from the transplanted organ.

Third, gestational tumors develop in patients with diverse histories and backgrounds, which might help to correlate reticulate evolution, should it be found, with epidemiological factors, including infections, and indicate that the uncovered phenomena are not limited to the unusual and rare group of transplant patients.

Finally, choriocarcinoma cells retain the ability of the trophoblast, the cell of origin, to fuse [25], implying that at least one mechanism of reticulate evolution, hybridization, is at work in these cancers.

What puzzles could gestational tumors help to explain?

One is that a fraction of circulating tumor cells (CTC) in some cancer patients - and in melanoma patients most of CTC - carry markers of hematopoietic cells [30, 31]. This observation is consistent with the hypothesis that neoplastic cells become metastatic by hybridizing with hematopoietic cells [32], a possibility supported by studies in animal models [17] and consistent with recent studies in transplant patients [24]. Analyzing individual circulating tumor cells from choriocarcinoma patients [29] would allow one to determine unambiguously whether such hybrids exist. Although finding hybrids in gestational tumors would not automatically mean that these hybrids exist in other cancers, this finding might attract enough interest and talent to learn how to detect reticulate evolution involving syngeneic cells.

A related observation, made while analyzing tumors in transplant patients, is that a fraction of phenotypically *neoplastic* cells in some tumors are of bone marrow origin [22, 33, 34]. How common these cells, which I suggest calling “adopted cells”, are or whether they even exists is uncertain. If they do exist, we would need to reconsider our understanding of cancer development and to review treatment strategies, as adopted cells have a different, origin and evolutionary history from the bulk of the tumor and thus different properties. Gestational tumors are a suitable model to decide whether this review is needed.

The third observation that gestational tumors can help to explore is the fact that neoplastic and adjacent normal cells sometimes share genomic aberrations, including those that are considered oncogenic [35, 36]. These aberrations may arise in normal cells independently, but borrowing them from cancer cells through cell fusion was also suggested as a possible mechanism [36] that has been demonstrated in an animal model [20].

Finally, gestational tumors can help to test whether cancer cells borrow DNA from normal cells. Such pirating could explain the puzzling cases of mutation reversion reported in carriers of mutated *BRCA1* or *BRCA2* genes [37-40]. Normal cells of these patients carry both the mutant and the normal allele, but cancer cells usually retain only the mutant. The lack of functional BRCA1 or BRCA2 makes cancer cells particularly sensitive to DNA damaging drugs because these proteins participate in DNA repair. However, the majority of the cancers relapse and some of them regain BRCA activity, thus becoming drug resistant.

Remarkably, in 13 out of 20 reported cases the activity was restored because BRCA proteins reverted to their wild type sequence, while in the remaining cases the restored activity was due to additional, compensatory, mutations [37, 38]. Because the reversion to wild type has been observed only in patients, but not in cell lines [38-41], it is not impossible that the normal allele was borrowed from normal cells and then used either directly, or as a template to repair the mutant allele by gene conversion. Gestational tumors can help to test this hypothesis.

Overall, gestational tumors provide an opportunity to test several long-standing unorthodox hypotheses, which, should they prove to be correct, could open new venues for cancer understanding, treatment, and prevention.
